# Holo-2bRAD: A Hologenomic Method for High-Resolution Analysis of Coral Microbiomes During Bleaching

**DOI:** 10.3390/microorganisms14040840

**Published:** 2026-04-08

**Authors:** Zhuqing Wang, Cen Ma, Heng Huang, Shaowen Ke, Jia Lv, Jingjie Hu, Shi Wang, Zhenmin Bao

**Affiliations:** 1 Southern Marine Science and Engineering Guangdong Laboratory (Guangzhou), Guangzhou 511458, China; hujingjie@ouc.edu.cn (J.H.); swang@ouc.edu.cn (S.W.); 2 Fang Zongxi Center for Marine Evo-Devo, MOE Key Laboratory of Marine Genetics and Breeding, College of Marine Life Sciences, Ocean University of China, Qingdao 266100, China; macen150@163.com (C.M.); lvjia@ouc.edu.cn (J.L.); 3 Institute of Aquatic Biotechnology, College of Life Sciences, Qingdao University, Qingdao 266071, China; 4 Key Laboratory of Tropical Aquatic Germplasm of Hainan Province, Sanya Oceanographic Institution, Ocean University of China, Sanya 572024, China; huangheng@stu.ouc.edu.cn; 5 Sanya Coral Reef Ecology Institute, Sanya 572025, China; keshaowen988@163.com; 6 Sanya Oceanographic Laboratory, Sanya 572025, China

**Keywords:** coral bleaching, hologenome, holo-2bRAD, microbial community

## Abstract

Coral reefs are biodiversity hotspots increasingly threatened by climate-induced bleaching, yet profiling the coral holobiont—the host and its associated microbiota—remains technically challenging due to high host-DNA contamination (often >95%) and the lack of comprehensive reference databases. Here, we present holo-2bRAD, a type IIB restriction site-associated DNA sequencing approach. This method, strategically integrated with a meticulously curated hologenome database (comprising 404,946 microbial genomes and 56 coral-derived metagenome-assembled genomes), effectively overcomes overwhelming host contamination (~99%). We demonstrate its exceptional species specificity (99.92%) in profiling *Galaxea fascicularis* (Linnaeus, 1767; Order Scleractinia, Family Euphylliidae) holobionts across bleaching severities, thereby validating its technical feasibility. Leveraging this high-resolution tool, our hologenome analysis revealed significant restructuring of coral-associated microbiota during bleaching, where microbial shifts (e.g., depletion of beneficial *Thermoanaerobacterium thermosaccharolyticum* and enrichment of stress-responsive bacteria) correlated more strongly with bleaching phenotypes than host genetic variation. By providing cost-effective, multi-domain hologenome profiling at unprecedented resolution, holo-2bRAD offers a practical tool for investigating holobiont dynamics and developing microbiome-informed coral conservation strategies.

## 1. Introduction

Coral reefs, often termed the “rainforests of the sea”, are biodiversity hotspots that support approximately 25% of marine species [1]. However, they are increasingly threatened by climate-induced coral bleaching—a stress response characterized by the breakdown of symbiosis between the coral host and its dinoflagellate algae (Symbiodiniaceae), accompanied by dysbiosis of associated microbial communities [2,3,4]. Corals function as complex holobionts, that represents a complex, multi-domain functional entity, encompassing the coral animal host, intracellular photosynthetic dinoflagellates, and a diverse array of associated microorganisms, including bacteria, archaea, fungi, and viruses [5,6]. These microbial communities inhabit various anatomical niches within the coral, such as the surface mucus layer, gastrodermal tissues, and skeletal pores, playing critical roles in nutrient cycling (e.g., nitrogen, carbon, phosphorus), host immunity, and overall coral health [7,8,9,10]. The intricate interplay within this holobiont is fundamental to coral adaptation and resilience, and dysbiosis within these symbiotic relationships is a key driver of phenomena like coral bleaching [2,3,4]. Conversely, coral health status shapes microbial diversity and composition, creating a dynamic feedback loop that modulates holobiont resilience [10,11,12]. Understanding these microbial dynamics is therefore essential for elucidating coral energy metabolism, health maintenance, and the resilience of reef ecosystems [9,13].

Recent omics-based studies have significantly advanced our knowledge of coral-associated microbial diversity and function [14,15]. Recent studies based on amplicon sequencing and metagenomics have extensively investigated the relationship between large-scale bleaching and coral-related bacterial communities, revealing that coral bleaching is associated with shifts in bacterial community structure, including the loss of putatively beneficial taxa and the proliferation of opportunistic bacteria [16,17]. Metagenomic analyses have identified functional genes related to nutrient cycling, stress responses, and virulence that may influence holobiont fitness under environmental stress [18].

Despite these advances, critical gaps remain in resolving species-level microbial dynamics during bleaching and integrating multi-omics data into a holistic framework [14,15]. Traditional amplicon sequencing, while suitable for low-biomass samples, suffers from significant limitations including high bias, low resolution, and the inability to simultaneously detect bacteria, fungi, and archaea [19,20]. Metagenomics is a widely used approach for assessing microbial diversity, offering comprehensive insights into the taxonomic composition, functional genes, and metabolic pathways of microbiomes [21]. However, this method requires high-quality DNA samples and is not suitable for analyzing samples with high host contamination, which is particularly challenging for corals. This is because coral collection is strictly limited in many countries (as corals are protected species), and symbiotic samples often contain excessive host DNA [22]. Additionally, the reference databases currently used for metagenomic analysis remain highly fragmented and lack comprehensive, well-integrated datasets, which is also why the bioinformatics processing of shotgun sequencing data has not yet been standardized [23,24]. This limitation is particularly pronounced for symbiotic organisms, significantly constraining the accurate analysis and annotation of their unique functional genes [15]. These bottlenecks severely restrict the analysis of the microbial dynamics during coral bleaching.

Recently developed 2bRAD-M technology addresses these challenges by leveraging type IIB restriction enzymes to generate uniform 20–33 bp genomic tags, enabling species-resolved profiling even in highly degraded or host-contaminated samples (e.g., 1 pg DNA, 99% host contamination) [25,26]. Originally developed for host genotyping, 2bRAD-M adapts this method for microbial community profiling by targeting conserved restriction sites across prokaryotic and eukaryotic genomes [25]. Unlike traditional methods, 2bRAD-M captures bacteria, archaea, and fungi simultaneously, achieving accuracy comparable to shotgun sequencing at 1% of the cost. Its efficacy has been validated in diverse contexts, including shrimp gut microbiota analysis, where it identified pathogen suppression and probiotic enrichment under chitosan oligosaccharide treatment [27]. Genomes lacking unique 2bRAD tags—due to the absence of BcgI recognition sites or the production of non-unique tags that cannot be reliably distinguished—are excluded from analysis to ensure database specificity and accurate species-level resolution [25,26]. Recently, we developed a simple yet effective holo-2bRAD method for tracking hologenome dynamics in marine invertebrates, which enables comprehensive host–microbe association analyses [28]. These features make 2bRAD-M uniquely suited for coral studies, where microbial biomass is often minimal and host DNA dominates. This approach enables high-resolution, cost-effective detection of both dominant and rare taxa, including strain-level variations, while minimizing PCR bias. Despite its potential, 2bRAD-M has yet to be applied to coral holobiont studies, particularly in the context of bleaching.

The present study aims to construct a species-specific 2bTag database for coral-associated microorganisms and validate technical feasibility through comparative analysis of healthy and bleached coral samples. This work establishes a novel omics-level tool for deciphering coral microbial community structure and provides a foundation for advancing molecular diagnostics and monitoring in coral conservation and reef ecosystem management.

## 2. Materials and Methods

### 2.1. Sampling and DNA Extraction

Coral samples were collected in July 2024 from Yalong Bay, Sanya, Hainan Province, during a mass bleaching event affecting colonies identified as *Galaxea fascicularis* s.s. (Linnaeus, 1767; Order Scleractinia, Family Euphylliidae). All colonies included in this study were identified morphologically by coral taxonomic expertise prior to downstream analyses. Seawater temperature records obtained from local monitoring stations indicated that the average surface temperature during the sampling period was 31.5 °C, approximately 3–4 °C above the historical summer mean for this region. Colonies of *G. fascicularis* were visually inspected and classified according to their bleaching severity using a standardized coral health chart [29]. Colonies were categorized into three distinct groups: non-bleached (Group A), exhibiting normal pigmentation; mildly bleached (Group B), showing partial paling; and severely bleached (Group C), displaying extensive or complete loss of color. For each bleaching category, three colonies were selected as biological replicates. Only colonies with intact morphology and without visible signs of disease, tissue necrosis, or extensive mortality were included to ensure that microbial differences were primarily associated with bleaching status rather than other pathological conditions.

Coral fragments (approximately 10–15 cm^3^) were collected from large, established colonies by SCUBA diving using a hammer and chisel at depths of 4–6 m. The fragment size was chosen to provide sufficient biological material for DNA extraction and holo-2bRAD library preparation while minimizing disturbance to the coral colony, which is a common practice in coral microbiome sampling. It is important to note that these fragments were small portions of large, established coral colonies, rather than entire small colonies. Immediately after collection, coral fragments were rinsed with sterile seawater to remove loosely attached debris and transient microorganisms. Samples were then flash-frozen on dry ice in situ to preserve nucleic acid integrity and transported to the laboratory for further analysis. Seawater samples (2 L each) were collected from the same reef site at the time of coral sampling at Yalong Bay, Sanya, Hainan Province, using sterile bottles deployed at approximately 1 m above the coral colonies. Water samples were transported to the laboratory on ice and immediately filtered through 0.22 μm Sterivex filters (JinTeng, Tianjin, China). The filters were stored at −80 °C until DNA extraction. Each bleaching category included three independent coral colonies, which were treated as biological replicates for subsequent analyses. Furthermore, two additional coral samples were collected from the healthy coral group (Group A) to verify the stability and reproducibility of the entire experimental pipeline. All samples were named according to the following convention: Rep 1 and Rep 2, technical replicate coral samples used to assess reproducibility; FA 1–FA 3, healthy coral samples (Group A); FB 1–FB 3, mildly bleached coral samples (Group B); FC 1–FC 3, severely bleached coral samples (Group C); and YLW 1–YLW 3, seawater samples collected from Yalong Bay as environmental reference samples.

It is important to note that the “coral tissue” samples collected for DNA extraction were intended to represent the entire coral holobiont. This bulk sampling approach captures the genomic material from the coral animal host, its intracellular algal symbionts (Symbiodiniaceae), and the diverse microbial communities (bacteria, archaea, fungi) intimately associated with the coral tissues and surface mucus layer. Therefore, the extracted DNA reflects the collective genetic information of this integrated biological unit.

The total DNA of corals was extracted using the MagPure Soil DNA KF Kit (Transgen Biotech, Beijing, China) according to the manufacturer’s instructions. Agarose gel of 1% concentration was used to detect whether DNA was successfully extracted through electrophoresis (125 V, 20 min). Then, the concentrations and purity of each successfully extracted DNA sample were measured by NanoDrop (Thermo Fisher Scientific, Waltham, MA, USA). All DNA samples were stored at −20 °C until further use.

### 2.2. Coral Holo-2bRAD Library Construction

DNA extracted from coral tissues was used to construct holo-2bRAD libraries following the protocol previously developed by our research group [26,30]. All procedures, including digestion, adapter ligation and PCR amplification of ligated adaptors, were performed following Ma’s protocol [28]. Concurrently, the conventional 2bRAD libraries constructed by DNA samples from the seawater were set as a comparison group respectively. Quality control and concentration measurement were performed using the Agilent 2100 Bioanalyzer DNA 1000 Chip (Agilent, Beijing, China) and qPCR. The libraries were sequenced by using the Illumina NovaSeq 6000 System (PE150 mode; Novagene, Beijing, China).

### 2.3. Data Analysis

The raw sequencing reads were subjected to trimming and quality filtering through a systematic series of procedures, which included the removal of reads containing ambiguous nucleotide calls (N), extended homopolymer stretches exceeding 10 base pairs, and reads with a high proportion of low-quality bases (more than 20% of bases exhibiting a quality score below 10). Subsequently, the filtered high-quality reads were processed to extract 2bRAD tags that encompass BcgI recognition sites. These tags formed the foundation for downstream analyses, including microbial community profiling, host genotyping, and taxonomic annotation based on reference databases.

Binning of coral metagenomics data. To improve the comprehensiveness of the holo-2bRAD database, we conducted binning analysis on coral metagenomic sequencing datasets obtained from the National Center for Biotechnology Information (NCBI) website with the 13 BioProject (PRJNA525179, PRJNA687160, PRJNA545886, PRJNA504905, PRJNA595374, PRJNA1086834, PRJNA1086806, PRJNA1082227, PRJNA1082198, PRJNA1061506, PRJNA1056882, PRJNA1023010 and PRJNA1001615). For each sample, metagenomic reads were assembled utilizing the Megahit pipeline (https://github.com/voutcn/megahit; accessed on 15 December 2024). Subsequent metagenomic binning was performed on these assemblies employing Maxbin 2.0 [31]. Genome quality metrics, including completeness, contamination, and strain heterogeneity, were assessed using the CheckM v1.0.11 pipeline (https://github.com/Ecogenomics/CheckM; accessed on 5 February 2025) under default parameters. Quality scores were calculated for each metagenome-assembled genome (MAG), and only those MAGs meeting predefined criteria were kept for analysis (completeness was above 20%, contamination below 10% and the genome size is between 1 M and 6 M).

Database construction. Genome-wide single-nucleotide polymorphism (SNP) genotyping was performed on all coral samples to characterize host genetic variation, using the RADtyping (version 1.30) computational pipeline (https://github.com/jinzhuangdou/RADtyping; accessed on 10 December 2024) for the systematic processing of high-quality holo-2bRAD sequencing tags [32]. To ensure genotyping accuracy, prefiltered and quality-validated 2bRAD sequencing tags were first aligned to the *G. fascicularis* reference genome (GCA_948470475.1) with SOAP2 v2.21 short-read aligner with default mapping parameters [33]. After successful genome alignment, codominant SNP calling was conducted via the codom_calling.pl script integrated within the RADtyping pipeline, with all parameters maintained at default settings to ensure analytical reproducibility. For rigorous filtering of reliable variant loci, only holo-2bRAD sites exhibiting a sequencing coverage depth ≥ 4× were retained as high-confidence SNP genotypes; genotypes with coverage below this threshold were designated as undetermined and excluded from downstream genetic analyses. To further explore the genetic relationships among individual coral samples, phylogenetic reconstruction was performed using the neighbor-joining (NJ) method implemented in the PHYLIP v3.69 software package [34]. The resulting NJ tree topologies were imported into the Interactive Tree Of Life (iTOL) v5.6 online platform (https://itol.embl.de/; accessed on 5 January 2025) for interactive visualization, annotation, and the production of publication-quality phylogenetic figures.

Microbial composition analysis. To elucidate the structural and compositional dynamics of coral-associated microbial communities, the 2bRAD-M pipeline [25] (https://github.com/shihuang047/2bRAD-M; accessed on 2 March 2025) was used for hologenome-referenced taxonomic and quantitative analysis of holo-2bRAD sequencing data. First, high-quality holo-2bRAD tags from all coral and seawater samples were aligned to the custom-built *G. fascicularis* hologenome database (constructed as described above). Taxonomic classification of microbial sequences and quantification of taxon-specific relative abundances were then performed via the standardized 2bRAD_M_Pipeline.pl workflow, which enables simultaneous profiling of bacterial, archaeal, and fungal communities from complex holobiont samples. To characterize microbial community patterns across all study samples, R-based bioinformatics tools were used for multivariate analysis and visualization: the ggplot2 (version 3.5.0) package was applied to plot spatiotemporal accumulation patterns of microbial taxa, heatmap functions to visualize taxonomic abundance variations among samples, and Venn diagram packages to identify core and unique microbial taxa across sample groups. Furthermore, inter-sample species co-occurrence networks were constructed to explore potential symbiotic or competitive interactions among coral-associated microorganisms. All key microbial community diversity indices, including α-diversity metrics (Shannon-Wiener, Simpson, and Chao1 indices) and species richness estimates, were calculated using PAST version 3 [35] with standard ecological community analysis parameters. Analysis of species with differential abundance in microbial communities was performed using the DESeq2 (version 1.38.2) method [25,28,36].

## 3. Results

### 3.1. The Principle and Workflow of the Holo-2bRAD Approach

**Figure 1:**
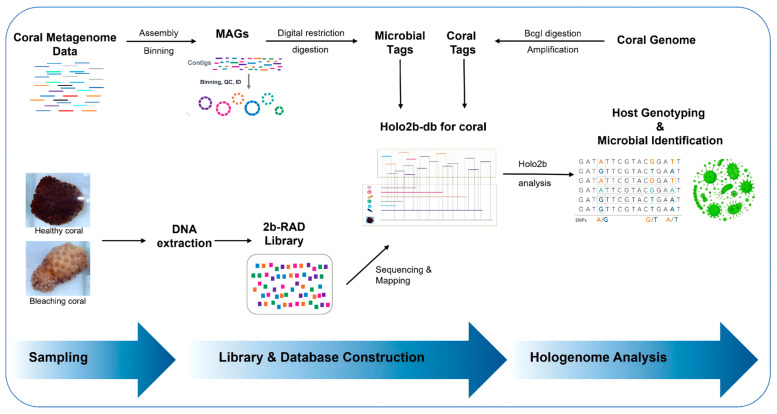
An overview of our holo-2bRAD approach for performing hologenome analysis in coral. Key procedural stages encompassed coral tissue sampling, simultaneous extraction of host and symbiont DNA, preparation of sequencing libraries, construction of reference hologenome databases, and bioinformatic analysis.

The practical application of the holo-2bRAD approach, which enables longitudinal analysis of the coral hologenome using a single sequencing library, relies on three essential conditions. First, the collection of coral samples, including both normal and bleached specimens for comparative analysis; second, the simultaneous extraction of composite genomic material containing genetic information from both the host and its symbiotic microbiota, a fundamental requirement for integrated hologenomic analysis; and last, the development of a customized reference database comprising host-specific and microbial taxon-specific restriction tags, enabling the precise bioinformatic discrimination of sequencing reads despite co-amplification within the library. An overview of the entire workflow is presented in Figure 1. Within the computational analysis workflow, the initial step involved constructing the coral hologenome database, which consists of microbial taxa-specific and host-specific 2bRAD tags. Detailed procedures for constructing this database are provided in the Section 2. Subsequently, raw sequencing reads underwent preprocessing, after which high-quality reads were mapped against the holo-DB to retrieve reliable reads that would be utilized for host genotyping and microbiota profiling. Ultimately, host-derived sequencing 2bRAD tags were genotyped via the RADtyping program for the acquisition of the host’s genome-wide genotypes. Conversely, microorganism-derived tags were employed to conduct analyses of microbial composition and relative quantification using the 2bRAD-M pipeline, consistent with the approach we previously applied in studies on scallops and shrimp [28].

### 3.2. Binning and Taxonomic Annotation of Coral Metagenomic Data

**Figure 2:**
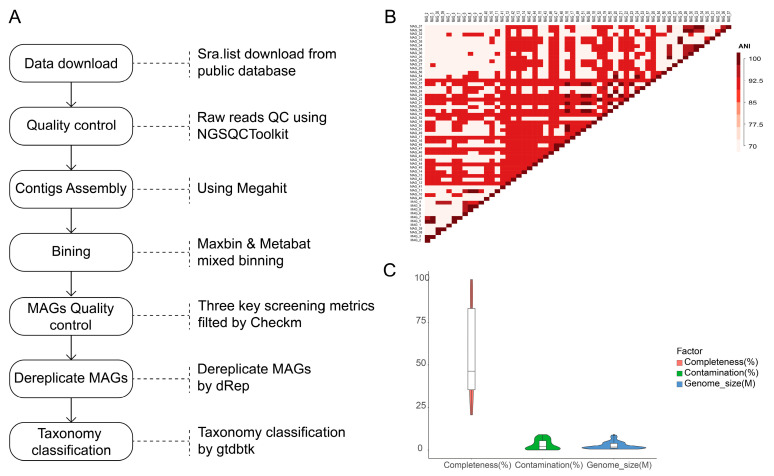
(**A**) Metagenomic binning workflow; (**B**) heatmap of MAG pairwise ANI values post-dereplication; (**C**) characterization of key metrics for the final MAG set (Completeness, Contamination, and Genome_size).

To further improve the comprehensiveness, representativeness and taxonomic coverage of the custom holo-2bRAD reference database, we integrated publicly available coral-associated metagenomic data and performed systematic metagenomic assembly and binning analyses. A total of 13 independent BioProjects deposited in the NCBI SRA database, encompassing 370 individual biosamples, were included in this integrative binning analysis (Figure 2). Metagenomic sequence assembly was conducted using the MEGAHIT pipeline, and MAGs were subsequently recovered from these assembled coral metagenomic datasets using the MaxBin 2.0 binning pipeline with default parameters. To eliminate redundant or highly similar genomic bins and ensure non-redundant representation at the species level, we calculated pairwise Average Nucleotide Identity (ANI) values across all recovered MAGs, and performed dereplication at a threshold of >97% ANI, which represents a widely accepted standard for delineating prokaryotic species (Figure 2B). The detailed workflow for metagenomic assembly, binning, dereplication and quality filtering is summarized in Figure 2A.

MAG quality assessment was performed using CheckM to estimate genome completeness and potential contamination. For subsequent analyses and database construction, only high-quality MAGs were retained: completeness > 20%, contamination < 10%, and genome size ranging from 1 to 6 Mb. After applying this rigorous quality-filtering procedure, we ultimately obtained 57 high-quality MAGs suitable for database integration. These qualified MAGs exhibited an average completeness of 57.84 ± 26.73%, an average contamination level of 3.14 ± 2.91%, and an average genome size of 2.55 ± 2.07 Mb (Table 1).

To determine the taxonomic identities and phylogenetic distribution of these binned microbial genomes, we performed standardized taxonomic annotation using the Genome Taxonomy Database Toolkit (GTDB-Tk) v2.4.0 [37]. In total, the metagenomic binning analysis yielded 57 high-quality microbial genome bins, including 56 bacterial genomes and 1 archaeal genome. These MAGs represented a broad taxonomic diversity, spanning 9 distinct phyla, 15 orders, and 24 genera, among which 5 MAGs were confidently classified to the species level (Figure 2C; Table 2). The recovery of these taxonomically diverse MAGs substantially expanded the depth and breadth of our holo-2bRAD reference database, thereby improving the accuracy of downstream microbial profiling and taxonomic assignment.

### 3.3. Construction of Hologenome Database (Holo-DB) in Coral

To ensure accurate and robust downstream analysis, a species-specific hologenome database (holo-DB) must first be constructed following our previously established protocol. This database contains high-confidence restriction enzyme-digested tags that enable simultaneous high-resolution taxonomic profiling of microbial communities and genotyping of the coral host.

To this end, a total of 404,946 microbial genomes were retrieved for database construction. This genome set included 57 non-redundant MAGs recovered from our metagenomic sequencing data, as well as 395,422 bacterial, 8004 archaeal, and 1505 fungal complete genomes sourced from NCBI RefSeq and EnsemblFungi databases. All collected genomes were then subjected to in silico restriction digestion using the type IIB restriction enzyme BcgI.

**Figure 3:**
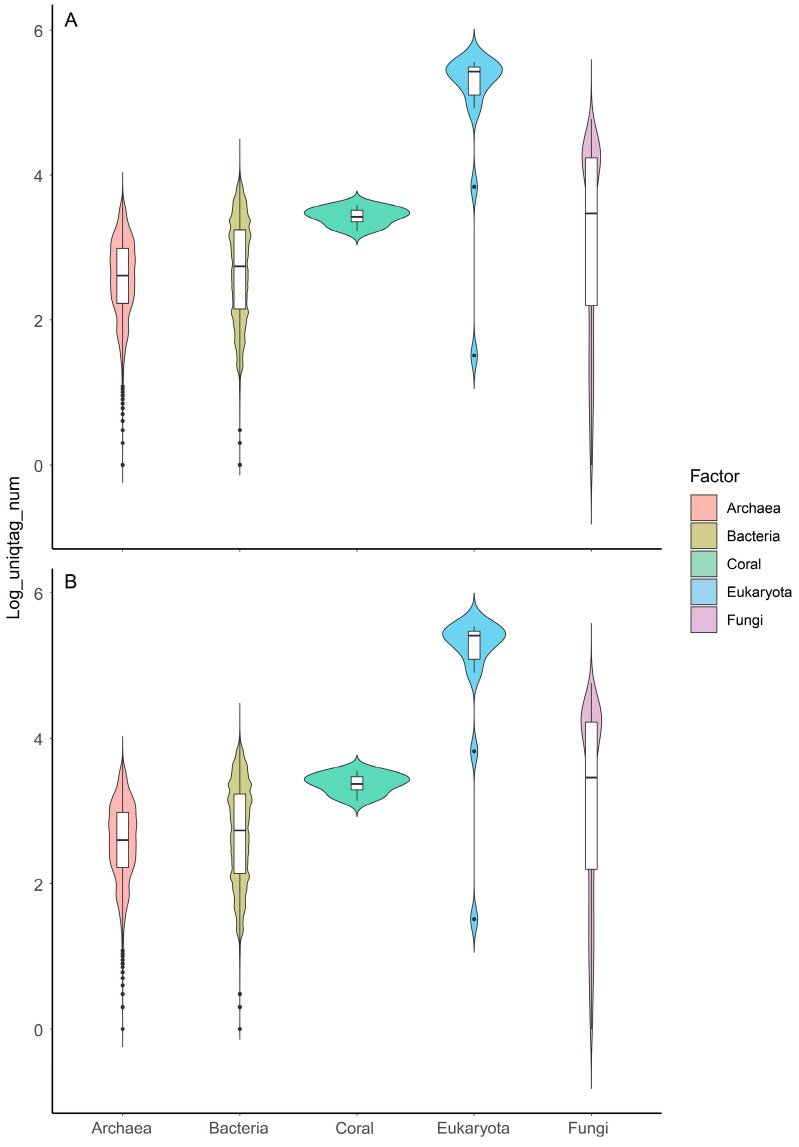
Distribution of theoretically existent specific-2bRAD tags at various kingdom levels of microbiota and *G. fascicularis*: (**A**) specific 2bRAD tags in both microbiota and coral; (**B**) specific 2bRAD tags in both microbiota and coral after a second round of redundancy removal.

Following the digestion step, a total of 404,628 genomes (representing 99.92% of the entire initial genome set) were retained; the remaining genomes were excluded owing to the absence of BcgI recognition sites or the generation of tags that were not unique and could not be reliably distinguished. These retained genomes were then integrated into the foundational holo-2bRAD database, with an average of 1264.955 tags generated per genome. From these generated tags, the target holo-2bRAD tags—defined as microbial taxa-specific DNA markers that exist as a single copy per genome—were selected for retention. Overall, across the various taxonomic ranks, a clear trend emerged: the higher the taxonomic level, the greater the number of available tags that could be retained (Figure 3A). At the kingdom level, minimal sharing of holo-2bRAD tags was observed among bacteria, fungi, and archaea; consequently, nearly all holo-2bRAD tags exhibited kingdom specificity. Specifically, phylum-specific, family-specific, and genus-specific holo-2bRAD tags constituted 99.70%, 99.50%, and 99.10%, respectively, of all theoretical holo-2bRAD tags produced from an individual microbial genome.

We further evaluated holo-2bRAD tag uniqueness across 86,902 species-level microbial taxa. Among the 51,834,062 total restriction fragments generated by BcgI digestion, approximately 99.92% existed as single-copy markers within their respective microbial genomes. Parallel in silico digestion was also performed on the *G. fascicularis* coral host genome. An initial set of 111,748 non-redundant host tags was obtained, which was further refined to 73,356 high-confidence unique host tags after strict second round of redundancy removal.

For final hologenome database (holo-DB) construction, microbial taxa-specific tags and host-unique tags were combined and subjected to a second round of redundancy removal. Through this step, only 658 coral-derived tags—accounting for 0.90% of all non-redundant tags—and approximately 8689 microbial genomes (representing 2.15% of the total microbial genome set) were removed (Figure 3B). The removal of these microbial genomes had negligible impact on taxonomic levels ranging from genus to phylum (e.g., impact rates of 0.01% to 0.1%). Notably, 86,902 species were retained during the database construction process (representing 99.89% of the total species); this observation indicates that the removal of the aforementioned microbial genomes exerted no significant effect at the species level.

Synthesizing these findings, there is minimal overlap in tags between the coral host and its associated microorganisms. This minimal overlap has virtually no influence on either the genotyping of the coral or the profiling of its associated microbiome. In turn, this outcome demonstrates the feasibility of performing hologenomic analysis using a single holo-2bRAD library.

### 3.4. Performance of Holo-2bRAD Analysis in Coral

To verify the reliability and stability of holo-2bRAD in coral hologenomic analyses, we constructed a set of technical replicate libraries (Rep 1 and Rep 2). The sequencing of these libraries yielded a comparable number of raw reads for the same species (Table 3). Following stringent quality control filtering, more than 96% of the sequenced reads were preserved as high-quality reads for downstream analysis. High-quality reads harboring the BcgI recognition sites were mapped to the coral hologenomic dataset. Across the two technical replicates, mapping rates to the coral host genome ranged from 19.99% to 20.06%, while the mapping rate to the associated microbial community remained stable at 0.06%, indicating highly consistent library construction and sequencing performance.

**Figure 4:**
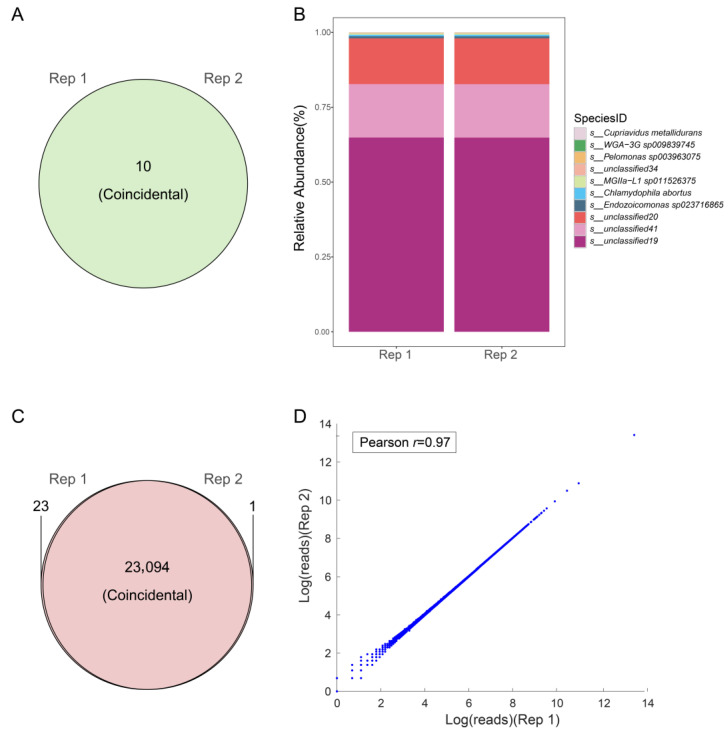
Performance of holo-2bRAD analysis in coral: (**A**) shared microbiological species between technical replicates of the coral; (**B**) stacked bar plot showing the microbial composition of the technical replicates; (**C**) shared 2bRAD tags of coral between technical replicates; (**D**) correlation of sequencing depth across 2bRAD tags between technical replicates (Pearson’s *r* = 0.97).

To evaluate the consistency of our methodology in characterizing the coral-associated microbial community, reproducibility was assessed. A high degree of similarity (100%) in the count of detected species was noted between the two technical replicates (Figure 4A). In the analysis of relative abundance, no significant differences were observed in the abundance of detected species between the two replicates (Figure 4B), as indicated by correlation analysis (Pearson’s *r* = 0.9995). This high reproducibility demonstrated the stable performance of the metagenomic analysis.

In addition to microbial profiling, we systematically assessed the performance of holo-2bRAD for coral host genotyping, focusing on four critical parameters: genome-wide tag coverage, genotype calling rate, genotyping concordance between technical replicates, and genotyping accuracy. Examination of genome-wide coverage of the detected 2bRAD sites revealed highly uniform coverage across all samples, with values of 77.28% and 77.29% for the two technical replicates, respectively. Both the genotype calls (showing 99.90% similarity) and the sequencing depth of each 2bRAD tag exhibited exceptional consistency between replicates. This high inter-replicate concordance was further supported by a Pearson correlation coefficient greater than 0.9706 (Figure 4C,D), collectively validating the robust stability and precision of holo-2bRAD for genome-wide host genotyping.

### 3.5. Hologenomic Analysis of Corals with Varying Bleaching Levels

**Figure 5:**
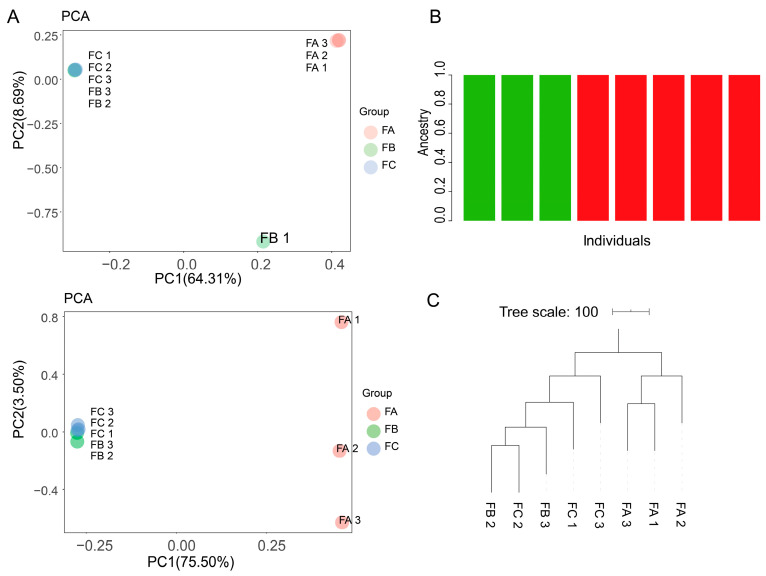
Genotypic profiling via RADtyping of corals with varying bleaching levels: (**A**) PCA clustering of SNPs (**upper**: pre-outlier removal; **lower**: post-outlier removal); (**B**) population stratification by ADMIXTURE; (**C**) neighbor-joining (NJ) phylogenetic tree using PHYLIP.

To further validate the practical applicability of holo-2bRAD in coral hologenomic research, we analyzed holo-2bRAD libraries generated from non-bleached, mildly bleached, and severely bleached *G. fascicularis* colonies, together with seawater reference samples collected during the same bleaching event (Table 3). Alignment to the custom hologenome database revealed clear differences in microbial mapping rates among coral samples with different bleaching severities (Table 4). RADtyping detected 3915, 3585, and 3440 SNPs in Groups A, B, and C, respectively, with 1619 shared across all groups. PCA of SNP data showed one outlier in Group B (attributed to individual factors); after exclusion, Group A formed a distinct cluster, while Groups B and C aggregated (Figure 5).

**Figure 6:**
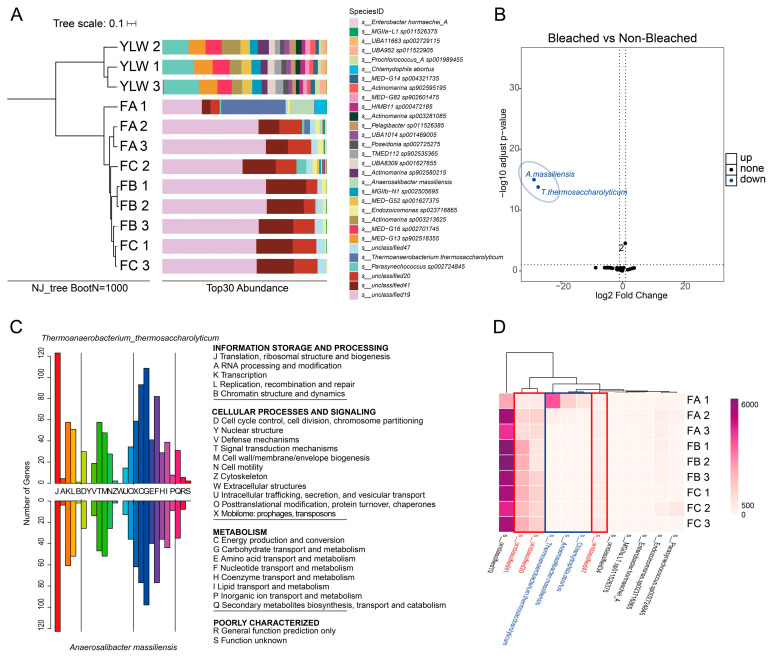
The microbiota in the three bleaching severity of corals: (**A**) clustering analysis and profiling stack column of coral and seawater samples at the species level; (**B**) DESeq2-based differential microbial taxa across experimental groups A and BC; (**C**) COG functional annotation of differential species genomes; (**D**) the relative abundance of detected species in 9 samples of coral at three bleaching levels. The three levels of bleaching: level A, light; level B, medium; level C, high.

Coral-associated microbiota across bleaching levels, together with seawater reference samples, were analyzed. Coral with lower bleaching severity had significantly higher microbial α-diversity (Shannon index, PERMANOVA, *p* = 0.003). Vallitaleaceae (42.38 ± 17.16%, 61.32 ± 2.48%, 53.63 ± 5.97%) and Pyrinomonadaceae (9.10 ± 3.66%, 22.51 ± 1.53%, 21.26 ± 1.71%) were dominant. Seawater microbiota showed distinct β-diversity from coral-associated communities (Bray–Curtis dissimilarity = 0.82, PERMANOVA, R^2^ = 0.46, *p* = 0.001; Figure 6A). DESeq2 identified two biomarkers: *Thermoanaerobacterium thermosaccharolyticum* (Group A-enriched, log2FC = −25.2, *p*-adj = 3.8 × 10^−6^) and *Anaerosalibacter massiliensis* (Group A-enriched, log2FC = −28.1, *p*-adj = 2.1 × 10^−8^; Figure 6B). COG enrichment analysis showed 68% of their genes mapped to translation (Category J; Figure 6C).

Three unresolved bacterial lineages (s_unresolved19, s_unresolved20 and s_unresolved41) exhibited dose-responsive enrichment with increasing bleaching severity (Figure 6D).

## 4. Discussion

### 4.1. Coral Holo-2bRAD: A Novel Tool for High-Resolution Hologenomic Profiling

This study pioneers the application of holo-2bRAD technology to coral holobiont research, overcoming two fundamental limitations in the field. By leveraging type IIB restriction enzymes (BcgI), this method resolves the critical challenge of high host-DNA contamination (up to 99%)—a ubiquitous obstacle in coral studies that has constrained prior metagenomic approaches. The generation of unique 20–33 bp tags enables precise discrimination between coral host genomes (73,356 host-specific tags) and microbial symbionts, achieving 99.92% specificity at the species level (validated by technical replicates with 99.90% genotyping consistency and Pearson’s *r* > 0.97). This capability allowed an accurate analysis of the coral microbial community, even in our severely bleached *G. fascicularis* samples, where host DNA dominated.

Furthermore, the hologenome concept posits coral specimens and their symbiotic microbiota function as a cohesive unit, with collective genomes (the hologenome) driving adaptation. However, progress in hologenomics has been hampered by fragmented databases and inconsistent taxonomic annotation [15]. Meanwhile, existing resources, such as the 170,000-microbe 2bRAD-M database, primarily focus on human or model organisms, neglecting marine symbionts. A key contribution of this work is the construction of a comprehensive and curated hologenome database (Coral holo-DB) for coral symbiosis research. Coral holo-DB integrates 395,422 bacterial, 8004 archaeal, and 1505 fungal reference genomes, along with 56 coral-derived MAGs and Symbiodiniaceae assemblies. This ensures broad coverage of coral-relevant microorganisms and provides a foundation for subsequent accurate taxonomic and functional analysis.

The database’s minimal cross-domain interference (<0.3% shared tags) and near-complete species-level tag retention (99.92% uniqueness) ensured the high-resolution detection of bacteria, archaea, fungi, and algae, thereby addressing key limitations of traditional amplicon sequencing (e.g., 16S sequencing). Crucially, holo-2bRAD achieves strain-level resolution at approximately 1% of the cost of shotgun metagenomics, as evidenced by reproducible identification of bleaching-sensitive taxa (e.g., *Thermoanaerobacterium thermosaccharolyticum* depletion) across replicates. The 2b-tag-DB enables cross-study comparisons via emerging tools like the Microbiome Search Engine (MSE), establishing a unified framework for coral hologenomics that surpasses human-centric databases (e.g., the 170,000-microbe 2bRAD-M database). Collectively, these advances establish holo-2bRAD as a practical and scalable tool for investigating microbial dynamics in coral holobionts.

### 4.2. Microbial Community Dynamics and Coral Bleaching

Hologenomic analysis revealed significant restructuring of microbial communities across bleaching severity gradients. Severely bleached corals exhibited a 21.5% reduction in alpha diversity (Shannon index, PERMANOVA *p* = 0.003), which is consistent with previous reports of stress-induced diversity loss [18,38]. Dominant families such as Vallitaleaceae (42.38–61.32% relative abundance) and Pyrinomonadaceae (9.10–22.51%) showed bleaching-stage-dependent fluctuations. Notably, taxa considered beneficial—*Thermoanaerobacterium thermosaccharolyticum* (log_2_FC = −25.2) and *Anaerosalibacter massiliensis* (log_2_FC = −28.1)—were significantly depleted in mildly bleached corals, while opportunistic bacteria proliferated. These shifts align with the “microbial dysbiosis” model of bleaching pathogenesis [16,17]. COG enrichment analysis revealed that 68% of differential taxa genes mapped to translational machinery (Category J), implying impaired protein synthesis may be a functional consequence of bleaching-associated dysbiosis. Additionally, three unresolved bacterial lineages (s_unresolved19, s_unresolved20, and s_unresolved41) exhibited dose-responsive enrichment that was correlated with bleaching severity. Although their ecological roles remain unclear, their consistent association with bleaching severity warrants further investigation.

While these findings highlight correlations between microbial community structure and bleaching phenotype, the observational design of this study does not permit causal inference. Future studies incorporating temporal sampling and experimental manipulations will be needed to disentangle cause from consequence. Moreover, benchmarking holo-2bRAD against traditional methods such as 16S rRNA amplicon sequencing represents an important next step. In previous work on scallops and shrimp, we demonstrated high concordance between 2bRAD-M and 16S sequencing at the genus level, with improved species-level resolution using 2bRAD-M [28,39]. For corals, we are planning a systematic comparison of holo-2bRAD with 16S amplicon sequencing and shotgun metagenomics across diverse sample types and bleaching conditions to further validate its performance and establish best practices for coral hologenome analysis.

### 4.3. Implications for Coral Conservation and Future Research Directions

The technical capabilities of holo-2bRAD have direct implications for coral conservation and monitoring. The method’s robustness with degraded samples and low microbial biomass (<0.06% microbial reads) makes it well-suited for large-scale reef health surveys, while its scalability supports longitudinal tracking of microbial succession during bleaching events.

From a conservation perspective, the observation that 1619 host SNPs were conserved across bleaching groups suggests that host genetic variation alone does not explain differential bleaching susceptibility. The finding supports the hologenome concept and challenges host-centric conservation paradigms, highlighting the potential value of microbiome-informed management strategies [40,41]. While direct microbial manipulation (e.g., probiotic inoculation) remains an active area of research, holo-2bRAD can provide the high-resolution data needed to identify candidate taxa and monitor intervention outcomes [42].

This holo-2bRAD technique also enables several practical applications: (1) Environmental DNA (eDNA) monitoring: Species-specific 2bRAD tag databases allow the non-invasive tracking of coral biodiversity and microbiome shifts from seawater samples, potentially enabling the early detection of bleaching-associated microbial signals. (2) Multi-omics integration: Combining host genotyping data with microbial taxonomic and functional profiles can help elucidate host–microbe interactions and identify molecular pathways involved in symbiosis breakdown. For example, integrating SNP data with COG profiles may reveal host–microbe modules linked to stress responses or metabolic dysfunction. In summary, the holo-2bRAD platform provides a practical, high-resolution approach for coral hologenome analysis, with the potential to shift reef conservation from passive restoration to proactive, data-driven resilience management. By enabling cost-effective, multi-domain profiling, this approach offers critical tools for understanding and mitigating climate change impacts on reef ecosystems.

### 4.4. Limitations and Future Directions

While holo-2bRAD represents a significant advancement in coral hologenomics, it is important to acknowledge certain limitations inherent to the approach. As a bulk DNA sequencing method, holo-2bRAD provides species-level taxonomic and genomic information for the entire holobiont community, but it lacks spatial resolution. This means it cannot precisely delineate the anatomical location (e.g., surface mucus layer, gastrodermal tissue, or skeletal pores) of specific microbial taxa within the complex coral structure. Future integration with spatially resolved techniques, such as fluorescence in situ hybridization (FISH), could complement holo-2bRAD by providing insights into the microhabitat distribution of identified microbes [43].

Finally, although we have constructed a comprehensive and meticulously curated hologenome database (holo-DB), the accuracy of taxonomic assignment and functional annotation is still ultimately dependent on the completeness and quality of the underlying reference genomes present within the database. While our holo-DB significantly expands coverage for marine symbionts, novel or poorly characterized taxa may still present challenges for precise identification [44]. Continuous expansion and refinement of such databases will further enhance the power of holo-2bRAD. Despite these limitations, holo-2bRAD provides an unparalleled, cost-effective platform for high-resolution hologenome profiling, laying a crucial foundation for future multi-omics integration and a deeper understanding of coral holobiont dynamics.

## 5. Conclusions

This study establishes holo-2bRAD technology with integrated coral-specific genomic resources for coral hologenomics analysis, resolving the persistent challenge of host-DNA contamination to enable species-level resolution in profiling holobiont dynamics. This methodology provides coral reef science with an enhanced toolkit to investigate holobiont interactions and advance the development of microbiome-informed conservation strategies.

## Figures and Tables

**Table 1:** Genomic information (e.g., completeness, contamination and genome size) of all MAGs.

ID	Completeness (%)	Contamination (%)	Genome Size (M)
MAG_1	43.51	5.53	1.04
MAG_2	39.14	7.74	1.21
MAG_3	41.89	6.25	1.32
MAG_4	38.20	8.57	1.86
MAG_5	35.37	3.85	1.03
MAG_6	26.66	9.02	1.11
MAG_7	38.41	6.15	1.03
MAG_8	35.32	5.13	1.05
MAG_9	41.88	7.44	1.08
MAG_10	92.89	6.41	2.15
MAG_11	34.75	1.96	1.03
MAG_12	97.99	1.59	5.64
MAG_13	98.88	2.24	4.29
MAG_14	89.12	3.26	4.20
MAG_15	45.03	9.06	2.37
MAG_16	97.60	0.20	4.75
MAG_17	83.08	4.89	5.09
MAG_18	92.41	2.30	4.12
MAG_19	71.81	3.33	5.81
MAG_20	22.98	1.90	1.69
MAG_21	98.00	0.60	4.79
MAG_22	96.53	5.60	5.22
MAG_23	100.00	0.00	4.83
MAG_24	82.71	5.20	2.34
MAG_25	34.80	7.92	1.02
MAG_26	40.44	3.93	1.08
MAG_27	34.69	4.51	1.03
MAG_28	43.66	3.14	5.14
MAG_29	35.73	1.62	1.29
MAG_30	27.58	7.02	1.55
MAG_31	20.69	0.00	1.02
MAG_32	46.36	1.01	1.00
MAG_33	32.56	3.34	1.01
MAG_34	48.63	7.45	1.33
MAG_35	21.98	0.88	1.08
MAG_36	26.72	4.10	1.48
MAG_37	44.10	1.77	1.12
MAG_38	56.25	0.00	1.04
MAG_39	63.58	1.27	1.23
MAG_40	68.93	1.51	1.71
MAG_41	33.62	6.90	1.31
MAG_42	99.36	0.32	3.35
MAG_43	36.21	0.00	1.39
MAG_44	87.01	0.94	3.37
MAG_45	82.22	1.05	2.81
MAG_46	95.48	7.60	6.00
MAG_47	81.88	2.05	3.98
MAG_48	55.33	0.00	2.21
MAG_49	22.28	0.00	1.05
MAG_50	41.53	0.00	2.22
MAG_51	53.33	0.00	2.20
MAG_52	73.27	1.93	3.38
MAG_53	60.09	0.27	1.28
MAG_54	70.18	0.00	1.83
MAG_55	94.80	0.00	4.39
MAG_56	25.86	0.00	1.02
MAG_57	93.80	0.13	4.43

**Table 2:** Taxonomic composition of MAGs.

Taxonomic Status	Number
Kingdom	2
Phylum	9
Class	10
Order	15
Family	22
Genus	24
Species	5

**Table 3:** Sequencing statistics of holo-2bRAD libraries of the coral and seawater samples.

	**Rep 1**	**Rep 2**	**FB 2**	**FB 3**	**FA 1**	**FA 2**	**FA 3**
Raw reads	5,490,761	5,509,821	9,128,371	6,589,171	6,489,176	6,871,027	6,389,172
High-quality reads	5,284,719	5,381,092	8,943,171	6,484,269	6,291,686	6,645,430	6,200,349
High-quality Reads Rate (%)	0.9625	0.9766	0.9797	0.9841	0.9696	0.9672	0.9704
High-quality tags with the restriction site	5,096,368	5,189,298	8,326,586	6,213,709	6,103,901	6,378,202	5,935,996
Mapping rate (Microorganism) (%)	0.0635	0.0635	0.4347	0.2832	0.3620	0.2876	0.3916
Mapping rate (Coral) (%)	19.9904	20.0612	20.1128	19.8233	19.7246	19.2826	18.9746
	**FC 1**	**FC 2**	**FC 3**	**YLW 1**	**YLW 2**	**YLW 3**	
Raw reads	5,881,723	7,387,120	6,428,197	6,982,178	6,382,919	7,109,283	
High-quality reads	5,698,265	7,109,071	6,236,780	6,710,129	6,179,854	6,960,801	
High-quality Reads Rate (%)	0.9688	0.9624	0.9702	0.9610	0.9682	0.9791	
High-quality tags with the restriction site	5,487,318	6,826,774	6,051,754	6,095,450	5,556,976	6,112,869	
Mapping rate (Microorganism) (%)	0.2724	0.2951	0.2742	13.9723	14.8156	6.7990	
Mapping rate (Coral) (%)	0.0020	0.0020	0.0020	--	--	--	

**Table 4:** Genotyping accuracy of coral.

	Homozygote	Heterozygote	All
Genotyped	655,711	8134	663,845
Same genotype	655,006	7830	662,836
Different, genotype	705	304	1009
Agreement (%)	99.89%	96.26%	99.85%

## Data Availability

The original contributions presented in the study are included in the article. Further inquiries can be directed to the corresponding authors.
